# Letter to Editor on Collagen‐Rich Extracellular Matrix and Postoperative Angiogenesis in Moyamoya Disease: The Need for Phenotype‐Stratified Transcriptomic Analysis

**DOI:** 10.1111/jcmm.71400

**Published:** 2026-10-11

**Authors:** Emaan Rani, Mirza Faaz Baig

**Affiliations:** ^1^ Rawalpindi Medical University Rawalpindi Pakistan; ^2^ Post Graduate Medical Institute Ameer ud Din Medical College Lahore Pakistan

**Keywords:** angiogenesis, cerebral revascularization, cerebrovascular disease, collagen‐rich extracellular matrix, extracellular matrix, gene expression, Moyamoya disease, postoperative angiogenesis, RNA sequencing, transcriptomic profiling

Dear Editor,

1

We have read the article titled ‘Transcriptomic Profiling Associates Collagen‐Rich Extracellular Matrix With Postoperative Angiogenesis in Moyamoya Disease’ by Chen et al. with great interest [1]. The authors deserve praise for their creative application of transcriptome‐wide RNA sequencing to temporal muscle tissue to uncover the crucial role of extracellular matrix (ECM) remodelling and collagen deposition in postoperative collateral vessel formation after indirect revascularization. A few drawbacks should be mentioned, however.

First, all the paediatric and adult patients with Moyamoya disease (MMD) are grouped together in the study cohort without stratification by clinical disease stage (e.g., Suzuki stage or hemodynamic stage) or age‐dependent angiogenic ability. In addition, as a natural trait of paediatric patients they have a much higher ‘native’ capacity for neoangiogenesis and collateral development than adult patients, irrespective of whether ECM is expressed. In addition, the endogenously generated differential angiogenic signals are associated with advanced disease stage and severe basal hypoperfusion. The collagen‐rich transcriptomic signature could be confounded by an age‐specific baseline angiogenic response or a drive towards ischemia in patients rather than a shared mechanism of surgical success, as the analysis of a mixed cohort lacks control of stage progression and age. As shown in Related Article, indirect revascularization efficacy is intrinsic to the exquisite balance between disease stage progression and age‐associated neoangiogenesis potential, suggesting that different patient subsets have different collateral formation [2]. Future studies are needed to study age‐matched groups (paediatric and adult separately) and to correct multi‐variable transcriptomic models for pre‐operative Suzuki staging and cerebrovascular reserve baseline.

Secondly, the investigation does not include a comparison or control group that underwent the direct bypass procedure or a combined bypass procedure (e.g., STA‐MCA bypass with encephaloduroarteriomyosynangiosis [EDAMS]). Direct bypass restores hemodynamic function immediately, changing the shear stress and metabolic function in adjacent tissues. Indirect bypass, on the other hand, is completely vascularized by tissue‐level angiogenesis. The results from this study cannot be generalised across different bypass techniques because of the absence of a comparative tissue sample for combined or direct bypass. As outlined previously in Related Article, the clinical and angiographic results of any combined bypass surgery are significantly different from those of any standalone indirect procedure, as is the local tissue perfusion pressures [3]. The addition of tissue controls from patients that have combined direct–indirect bypass would allow to separate the expression patterns of ECM molecules that are direct–indirect bypass specific from those that are part of the general tissue healing process under different flow conditions in future transcriptomic studies.

Thirdly, the study groups MMD into binary collateral outcome categories (Good vs. Poor using the Matsushima grading system), while the category for primary clinical presentation (ischemic vs. hemorrhagic) is not taken into account. Ischemic and hemorrhagic MMD have different underlying pathophysiological mechanisms, underlying vessel wall inflammation, and postoperative trajectory of collateral formation. Microvascular fragility and altered expression of matrix metalloproteinases are a common component of the picture of hemorrhagic MMD and are directly related to temporal muscle tissue biology and angiogenesis after the procedure. The unchecked accumulation of both phenotypes will lead to an unchecked mixing of phenotype‐specific molecular drivers of collateralization. Collateral formation dynamics and angiographic response following indirect bypass are different depending on the aetiology (hemorrhagic vs. ischemic). Subgroup analyses of the ischemic and hemorrhagic patients should be undertaken by researchers to determine whether the collagen‐rich ECM signature is similar in both presentations [4].

To summarise, Chen and colleagues have given a much‐needed molecular underpinning of the relationship between extracellular matrix remodelling and collagen deposition in temporal muscle tissue with subsequent successful postoperative collateralisation in Moyamoya disease. However, angiogenic potential, disease stage, surgical approach variability and clinical phenotype (ischaemic vs. hemorrhagic) are still important factors to consider. Therefore, these parameters need to be addressed in multi‐centre phenotype‐stratified prospective cohorts to make these transcriptomic discoveries relevant to targeted therapeutic tools that will improve surgical revascularization outcomes.

## Author Contributions

**Emaan Rani:** methodology, resources, writing – original draft. **Mirza Faaz Baig:** supervision, writing – review and editing.

## Funding

The authors have nothing to report.

## Ethics Statement

The authors have nothing to report.

## Conflicts of Interest

The authors declare no conflicts of interest.

## Data Availability

The authors have nothing to report.
